# Night-break treatment with blue and green lights regulates erect thallus formation in the brown alga *Petalonia fascia* (KU-1293)

**DOI:** 10.3389/fpls.2024.1500947

**Published:** 2025-01-15

**Authors:** Yuya Maegawa, Fumio Takahashi, Tsunami Hiyama, Shinya Yoshikawa

**Affiliations:** ^1^ Faculty of Marine Science and Technology, Fukui Prefectural University, Obama, Fukui, Japan; ^2^ Faculty of Pharmaceutical Sciences, Toho University, Funabashi, Chiba, Japan

**Keywords:** photomorphogenesis, blue light receptor, green light receptor, phaeophyceae, photoperiodism

## Abstract

In this study, we investigated the photoperiodic responses regulating erect thallus formation in *Petalonia fascia* (KU-1293). We found that, through critical day length analysis and night break treatment culture experiments, *P. fascia* formed erect thalli under short-day conditions, indicating a genuine photoperiodic response. The critical day length for this morphological change was 10–11 h. Notably, night-break treatment with blue light (BL) and green light (GL) inhibited, whereas that with red light (RL) enhanced erect thallus formation. *P. fascia* was more sensitive to BL than to GL, requiring approximately 100-times less light to exhibit similar effects. Furthermore, promotion of erect thallus formation by RL was not negated by far-red light, suggesting the presence of a novel RL receptor distinct from the classical phytochrome system in terrestrial plants. These findings highlight the complexity of light wavelength interactions in regulating photoperiodic responses and suggest the presence of unique photoreceptors for day-length perception and erect thallus formation in *P. fascia*.

## Introduction

Brown algae are among the major primary producers in coastal ecosystems. Similar to land plants, alternating generation and morphology in response to physical conditions, such as light, temperature, and dehydration, are important for the environmental adaptation of immobile brown algae. Seasonal changes are among the most influential environmental factors for brown algae. They sense the seasonal transitions based on day length, regulate physiological activities, and alternate generations. Many studies have investigated photoperiodism in land plants, with most focusing on morphological formation, such as flower bud formation and fruit ripening (Thomas and Vince-Prue, 1997). The response of an organism to day length is known as photoperiodism. Many studies have reported photoperiodism in animals, fungi and terrestrial plants (Nelson et al., 2010). In angiosperms, night-break (NB) treatment, which involves a short period of light irradiation in the middle of the dark period, is among the most useful methods for photoperiodism analysis (Thomas and Vince-Prue, 1997). As the day length affects the total amount of photosynthetic products involved in growth and physiological functions, NB treatment helps in determining whether certain morphogenesis is induced by a photoperiodic response (photoperiodism) (Parker et al., 1950). Moreover, NB treatment with monochromatic light helps to identify the photoreceptors controlling photoperiodic responses (Parker et al., 1945). In land plants, photoperiodically controlled responses are dependent on photoreceptors rather than photosynthesis. Analysis of the NB action spectrum revealed that photoperiodicity in short-day (SD) plants is regulated by the perception of phytochromes to red light (RL) and far-red light (FR) (Thomas and Vince-Prue, 1997).

To date, only physiological experiments have been conducted on brown algae, with only a few studies investigating photoreceptors using molecular biology techniques. Photomorphogenesis controlled by the photoperiod has been reported in several genera of brown algae, including erect thallus formation in *Scytosiphon lomentaria* (Dring and Lüning, 1975a), gamete formation in *Sphacelaria rigidula* (Ten Hoopen et al., 1983), sorus formation in *Saccharina* and *Undaria* (Lüning, 1986, Lüning, 1988; Pang and Lüning, 2004), stem elongation in *Sargassum muticum* (Hwang and Dring, 2002), and receptacle initiation in *Ascophyllum nodosum* and *Sargassum horneri* (Terry and Moss, 1980; Yoshikawa et al., 2014). These reports suggest that the photoperiod is an essential environmental factor affecting the seasonality of brown algae and that brown algae possess photoreceptors to perceive day length. However, the specific receptor molecules involved in photoperiodism in brown algae remain elusive. This is due to the scarcity of studies analyzing the wavelengths controlling photoperiodism in brown algae (Dring and Lüning, 1975a; Terry and Moss, 1980; Yoshikawa et al., 2014) despite the important effect of light quality on photoperiodic responses and photoreceptor molecules.

Analysis of the effect of NB treatment on erect thallus formation in *S. lomentaria* revealed that the photoperiodic responses are regulated only by blue light (BL) (Dring and Lüning, 1975a). In contrast, NB treatment with RL and BL induces receptacle formation in *A. nodosum* (Terry and Moss, 1980). We previously showed that NB treatment with green light (GL) and BL effectively initiates receptacle formation in *Sargassum horneri* (Yoshikawa et al., 2014). However, whether brown algae have common photoreceptors associated with photoperiodism remains unclear. Moreover, differences in light quality related to photoperiodism hinder the comprehensive understanding of photoperiodism in brown algae. To determine the photoreceptors controlling photoperiodism in brown algae, we focused on the photoperiodic responses affecting erect thallus formation in *Petalonia fascia* in this study. A previous study reported that *P. fascia* forms an erect thallus from a discoid thallus under SD conditions (Roeleveld et al., 1974), suggesting that the erect thallus formation is a photoperiodic response in *P. fascia*. However, detailed physiological analysis of the photoperiodism of *P. fascia* has not yet been performed. Here, we analyzed the effects of various light intensities and qualities on erect thallus formation and photoperiodic responses in *P. fascia*.

## Materials and methods

### Materials

A strain of *P. fascia* (KU-1293) was obtained from the Kobe University Macro-Algal Collection.

### Culture conditions

For subculture, *P. fascia* was cultured in the ESI medium (Tatewaki, 1966) without Tris at 15°C under 30 µmol photons m^-2^ s^-1^ fluorescent lamp light (FL20SSEX-D; Toshiba, Tokyo, Japan) and long-day (LD) conditions (16 h light and 8 h dark). The same temperature conditions and medium were used in all experiments. Thalli were pinched off from the subculture and incubated in a flask with 100 mL medium for two weeks as a pre-culture.

### Light–dark cycle and NB treatment

To determine whether erect thallus formation depends on photoperiodism, we investigated erect thallus formation using critical day length and NB treatment. The pre-cultured discoid thalli of *P. fascia* were statically cultured in a 24-well plate (14530; Thermo Fisher Scientific, MA, USA) under 30 µmol photons m^-2^ s^-1^ fluorescent lamp light and various light: dark regimes from 9:15 h to 16:8 h. After 24 d, erect thallus formation rate was calculated by dividing the number of erect thalli formed in a well by the amount of chlorophyll a (µg) in the same well. The number of erect thalli within the well was counted using a stereomicroscope (SZX-10; Olympus, Tokyo, Japan) equipped with a digital camera (DP12; Olympus). Chlorophyll a content of the well was calculated from the absorbance values using 90% acetone (Jeffrey and Humphrey, 1975). In NB treatment, 1-h light was provided 7 h after the start of the dark period under short-day conditions (SD, 10 h light and 14 h dark). The following light sources were used for NB: white light (WL), BL (LED illuminators ISL-150×150-BB LED illuminators; peak wavelength: 472 nm; full width at half maximum: 24 nm; CCS Inc., Kyoto, Japan), GL (QFC45-100PG; peak wavelength: 531 nm; full width at half maximum: 21 nm; ACETEC, Tokyo, Japan) with a sharp cut-off filter (SC52; Fujifilm, Tokyo, Japan), RL (ISL-150×150-RR; peak wavelength: 654 nm; full width at half maximum: 18 nm; CCS), and FR (ISL-150×150-FR; peak wavelength: 738 nm; full width at half maximum: 27 nm; CCS). Intensity of each light source was adjusted by changing the distance between the light source and plate or using an ND filter (ND-0.1; Fujifilm). Light intensity in NB treatment with BL, GL, and RL was 10 µmol photons m^-2^ s^-1^. Light intensity in NB treatment with FR was 50 µmol photons m^-2^ s^-1^. Emission spectra (**Supplementary Figure 1**) and photon fluence rates of the light sources used for NB treatment were measured using the Light Analyser LA-105 (Nippon Medical & Chemical Instruments Co., Ltd., Osaka, Japan). To assess the difference in sensitivity to BL and GL, effects of different durations (60–3600 s) of NB treatment with various intensities (0.1–10 µmol photons m^-2^ s^-1^) of BL and GL on erect thallus formation were examined (**Supplementary Table 1**).

Significant differences among groups were analyzed using one-way analysis of variance, followed by the Bonferroni test.

### *In vivo* absorption spectrum

The *in vivo* absorption spectrum of discoid thallus was acquired with Spectrophotometer U-3300 (Hitachi, Ltd., Tokyo, Japan). Thalli were suspended in the culture medium and placed in a 10-mm path-length cuvette. Absorbance was recorded at 1-nm intervals across 400 to 700 nm wavelengths. To obtain the relative absorption spectrum, the absorbance at 437 nm, which showed the highest value, was normalized to 1, and the absorbance at 700 nm, which showed the lowest value, was normalized to 0.

## Results

### Erect thallus formation under SD conditions

**Figure 1:**
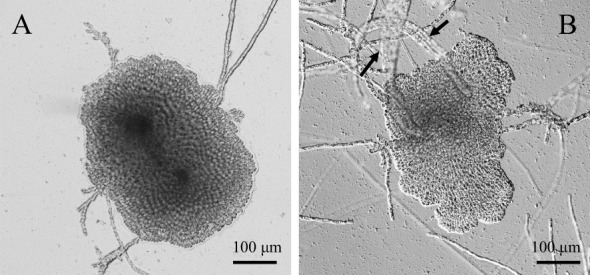
Photoperiod-dependent changes in the morphology of *Petalonia fascia*. **(A)**. Discoid thalli under long-day (LD) conditions. **(B)**. Erect thalli (arrows) produced on the discoid thalli under short-day (SD) conditions.

Consistent with previous reports (Roeleveld et al., 1974), almost all thalli of *P. fascia* were discoid (disc thalli) under LD (**Figure 1A**), and erect thalli were formed only under SD conditions (**Figure 1B**). Erect thalli grew directly from the discoid thalli. Erect thallus formation was low under LD conditions and high under SD conditions.

### Critical day length for erect thallus formation

**Figure 2:**
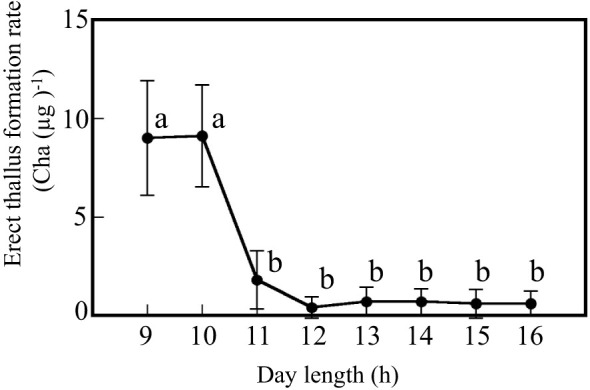
Critical day length for erect thallus formation in *P. fascia*. After 24 d of culture, erect thallus formation rates were evaluated. Bars indicate the standard deviations. Letters indicate the significant means determined using the Bonferroni test (P < 0.01; n = 24).

Discoid thalli were cultured for various day lengths to determine the critical day length for erect thallus formation (**Figure 2**). When the discoid thalli were cultured with a day length over 11 h, only a few erect thalli were formed. Erect thallus formation rate with the 11-h day length was 1.8 Cha (µg)^-1^. No significant differences in erect thallus formation rates were observed from the 11- to 16-h day lengths. In contrast, erect thallus formation rates with 9- and 10-h day lengths were 9.0 and 9.1 Cha (µg)^-1^, respectively, which were significantly higher than that with 11-h day length (P < 0.01; **Figure 2**). Erect thallus formation rate with the 10-h day length was approximately four-times higher than that with the 11-h day length. These results suggest that the critical day length for erect thallus formation is between 10 and 11 h in *P. fascia*.

### NB treatment for erect thallus formation

**Figure 3:**
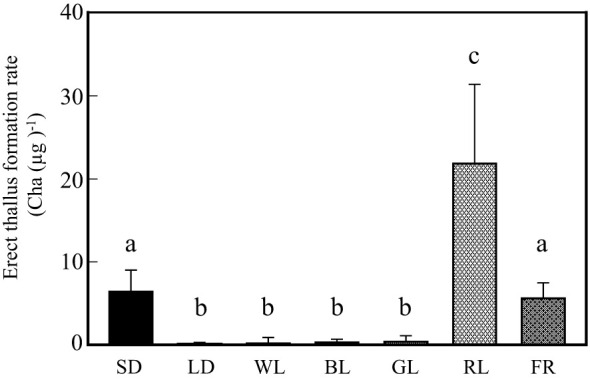
Effects of night-break treatment with different wavelengths of light on erect thallus formation in *P. fascia*. Thallus of *P. fascia* underwent night-break treatment for 1 h with white light (WL), blue light (BL), green light (GL), red right (RL), and far-red light (FR) 7 h after the start of the dark period. Erect thallus formation rates under LD and SD conditions are shown. After 24 d of culture, erect thallus formation rates were evaluated. Bars indicate the standard deviations. Letters indicate the significant means determined using the Bonferroni test (P < 0.05; n = 24).

Next, NB experiments were performed to confirm that erect thallus formation is influenced by photoperiodism and identify the effective wavelength to control photoperiodism. Erect thallus formation rate was 0.2 Cha (µg)^-1^ after 1-h NB treatment with WL, which was almost the same as that under LD conditions (0.1) but significantly lower than that under SD conditions (6.4 Cha (µg)^-1^; P < 0.01; **Figure 3**). These results indicate that erect thallus formation in *P. fascia* is regulated by photoperiodism. Next, we investigated the effects of various wavelengths of NB treatment on the erect thallus formation rate. As shown in **Figure 3**, NB treatment with BL and GL inhibited erect thallus formation, leading to erect thallus formation rates of 0.3 and 0.4 Cha (µg)^-1^, respectively (**Figure 3**). Erect thallus formation rate under these conditions was significantly lower than that under normal SD conditions (P < 0.01). Notably, erect thallus formation rate was higher after NB treatment with RL (21.8 Cha (µg)^-1^) than under SD conditions (P < 0.05; n = 24; **Figure 3**), being more than twice the value under SD conditions. However, no significant difference was observed in the erect thallus formation rate after NB treatment with FR (5.6 Cha (µg)^-1^) and under SD conditions (P > 0.05; n = 24).

**Figure 4:**
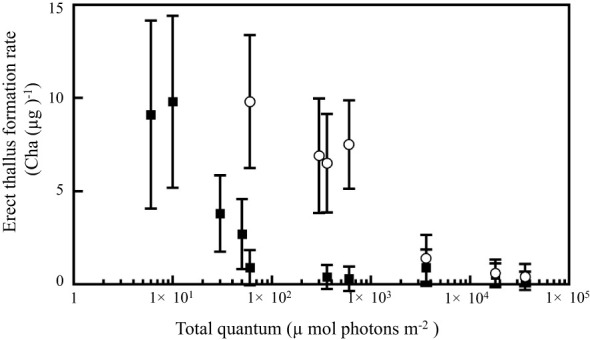
Effects of the total quantum of night-break treatment with BL and GL on erect thallus formation in *P. fascia*. Open circles and closed squares indicate the effects of GL and BL, respectively. After 24 d of culture, erect thallus formation rates were evaluated. Bars indicate the standard deviations.

To investigate the sensitivity of day-length perception to BL and GL in *P. fascia*, we analyzed the relationship between total quantum irradiation (**Supplementary Table 1**) and erect thallus formation. Erect thallus formation rate decreased with increasing photon quantities in NB treatment with BL and GL. However, erect thallus formation in *P. fascia* showed very high sensitivity to BL rather than to GL (**Figure 4**; **Supplementary Figure 2**). More than 30 µmol photons m^-2^ (1.5 µmol photons m^-2^ s^-1^, 20 s) irradiation in NB treatment with BL significantly decreased the erect thallus formation rate compared to that under SD conditions (**Supplementary Figure 2**). At photon quantities < 600 µmol photons m^-2^, effect of GL on erect thallus formation was significantly weaker than that of BL (P < 0.01; **Figure 4**; **Supplementary Figure 2**). More than 3600 µmol photons m^-2^ (1 µmol photons m^-2^ s^-1^, 3600 s) irradiation was needed to achieve the same effect with BL. These results revealed that the sensitivity to BL was almost 100-times higher than that to GL for erect thallus formation in *P. fascia*.

To estimate the photosynthetically utilized radiation (PUR) absorbed primarily by photosynthetic pigments, we obtained *in vivo* absorption spectra of *P. fascia* from 400–700 nm (**Supplementary Figure 3**). The relative absorbance values for BL (472 nm)and GL (532 nm) were 0.84 and 0.55, respectively, indicating that blue light is absorbed approximately 1.5 times more than green light, primarily by photosynthetic pigments. Considering the PUR absorption (**Supplementary Figure 3**) and the light intensities that inhibit erect thallus formation (**Figure 4**; **Supplementary Figure 2**), it is estimated that the sensitivity to BL in erect thallus formation is about 150 times higher than to GL.

### Verification of photoreversibility

**Figure 5:**
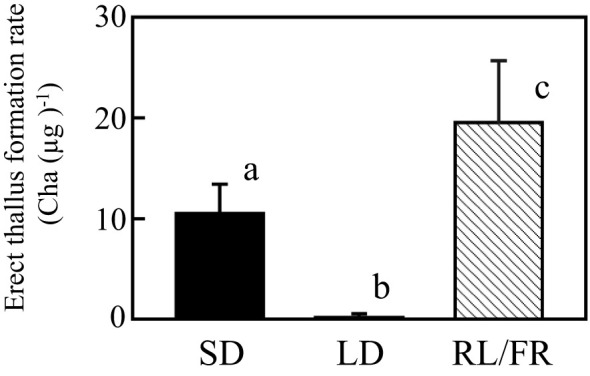
Effect of FR irradiation immediately after RL irradiation on erect thallus formation in *P. fascia*. Effect of 1-h night-break treatment with RL, followed by 1-h FR irradiation on erect thallus formation was examined. Erect thallus formation rates under LD and SD conditions are shown. After 24 d of culture, erect thallus formation rates were evaluated. Bars indicate the standard deviations. Letters indicate the significant means determined using the Bonferroni test (P < 0.01; n = 24).

In some angiosperms, phytochrome-mediated RL responses, such as seed germination and flowering, are cancelled by FR (Thomas and Vince-Prue, 1997). Therefore, in this study, we examined whether increased erect thallus formation by photoreceptors, such as phytochromes, in *P. fascia* was cancelled by NB treatment with FR. Notably, 1-h FR irradiation following NB treatment with RL did not cancel the effect of RL, and erect thallus formation rate was significantly higher than that under SD conditions (**Figure 5**; P < 0.01; n = 24).

**Figure 6:**
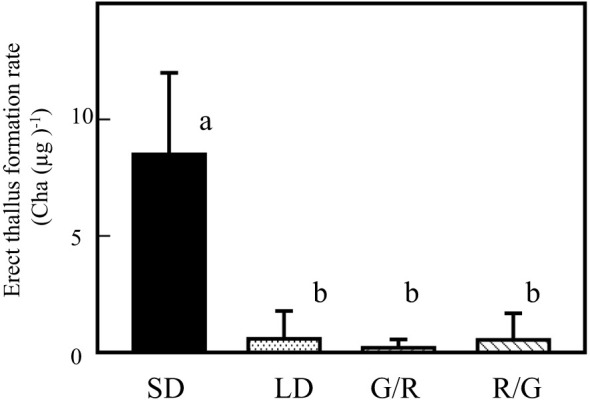
GL effects on *Petalonia fascia* are cancelled by RL and vice versa. Effect of 1-h night-break treatment with GL, followed by 1-h RL irradiation on erect thallus formation was examined (G/R). Effect of 1-h night-break with RL, followed by 1-h GL irradiation on erect thallus formation was examined (R/G). Erect thallus formation rates under LD and SD conditions are shown. After 24 d of culture, erect thallus formation rates were evaluated. Bars indicate the standard deviations. Letters indicate the significant means determined using the Bonferroni test (P < 0.01; n = 24).

Recent studies have focused on the biochemical analyses of diatom phytochromes. Phytochromes of diatoms and brown algae are reversible under GL and RL exposure (Rockwell et al., 2014). To assess the relationship between RL-induced erect thallus formation and photoperiodic response, effects of 1-h GL irradiation following NB with RL (G/R) and 1-h RL irradiation following NB with GL (R/G) were analyzed (**Figure 6**). Regardless of whether RL irradiation was performed before or after GL irradiation, only the effect of GL was observed. Erect thallus formation rates under LD, G/R, and R/G conditions were 0.6, 0.2, and 0.5 Cha (µg)^-1^, respectively, which were significantly lower than that under SD conditions (8.5 Cha (µg)^-1^; P < 0.01; n = 24). These results suggest that the effect of RL on erect thallus formation is limited to SD conditions. Collectively, all NB experiments revealed that *P. fascia* uses BL and GL to perceive day length.

## Discussion

Previous studies on the life cycle of *P. fascia* have reported erect thallus formation under SD conditions (Roeleveld et al., 1974). However, to date, no study has conducted physiological analysis to determine whether this phenomenon is controlled by a genuine photoperiodic response or the total light period. In this study, we demonstrated that erect thallus formation in *P. fascia* is regulated by photoperiodism.

Previous studies reported that the light quality affects photoperiodism in three brown algal species: *Scytosiphon lomentaria*, *Ascophyllum nodosum*, and *Sargassum horner*i (Dring and Lüning, 1975a; Terry and Moss, 1980; Yoshikawa et al., 2014). These reports are consistent with our findings on BL-controlled photoperiodism. Therefore, BL is possibly a common mechanism for day-length perception in brown algae. Here, 10 s of dim light NB treatment with BL (total quantum: 30 µmol photons m^-2^) inhibited erect thallus formation, indicating the high sensitivity of *P. fascia* to BL. This effect is comparable to that observed in *S. lomentaria* after NB treatment with BL (total quantum 20 µmol photons m^-2^) (Dring and Lüning, 1975a).

Notably, effects of NB treatment with GL on *P. fascia* in this study were clearly different from those in previous studies on *S. lomentaria*. Dring and Lüning reported that the action spectrum for erect thallus formation in *S. lomentaria* had no effect in the wavelength range of 501–724 nm (Dring and Lüning, 1975a cf.; **Figure 5**). Differences between our results and previous findings on the effects of GL may be due to differences in the total quantum rather than differences in the experimental organisms. Here, NB treatment with 1 µmol photons m^-2^ s^-1^ (60 min; total quantum 3600 µmol photons m^-2^) GL inhibited erect thallus formation in *P. fascia*. In contrast, Dring and Lüning reported that NB treatment with 1 µmol photons m^-2^ s^-1^ (1 min; total quantum 60 µmol photons m^-2^) GL has no effect on erect thallus formation. As the total quantum of GL irradiation in this study was 60-times higher than that in the study by Dring and Lüning, GL effectively suppressed erect thallus formation in *P. fascia*.

The light intensity and duration of GL exposure used in this study were comparable to those observed in the natural growth environment of *P. fascia*. GL intensity of 1 µmol photons m^-2^ s^-1^ may cause disc thallus formation in *P. fascia*. As GL is more prominent than BL in phytoplankton-rich environments (Jaubert et al., 2017), the mechanism to measure day length using GL is helpful for the environmental adaptation of *P. fascia*. BL-and GL mediating photoperiodic responses have been reported in *S. horneri* (Yoshikawa et al., 2014). However, only a few studies have investigated the spectral quality related to photoperiodism in brown algae. Moreover, whether most brown algae use GL in the same manner as BL to measure day length remains unclear. Future studies should investigate the photoperiodicity and light quality in other brown algal groups to determine whether photoperiodicity control via GL is a shared characteristic among brown algae.

The BL photoreceptors, aureochromes and cryptochromes, are possible photoreceptors controlling photoperiodism in *P. fascia* based on the identified photoreceptor genes in the brown alga *Ectocarpus siliculosus* (Cock et al., 2010).

Aureochromes are BL receptors unique to stramenopiles. Although their functions in brown algae remain unknown, they are involved in branch formation in the xanthophyte *Vaucheria frigida* (Takahashi et al., 2007) and cell division in diatoms (Huysman et al., 2013). Recent studies in diatoms have reported that aureochromes are involved in light-dependent responses, including photoprotection (Zhang et al., 2024) and the regulation of the circadian clock (Madhuri et al., 2024). As aureochromes are composed of a BL-sensing LOV domain and b-zip domain that binds to DNA, they perform dual functions: photoreception and regulation of gene expression (Takahashi et al., 2007; Takahashi, 2016). Therefore, aureochromes may be the key photoreceptors involved in photoperiodism and gene expression control in brown algae. However, as aureochromes exhibit an absorption spectrum similar to that of typical flavin proteins and the absorption does not include the GL region (> 500 nm) (Hisatomi et al., 2013), involvement of aureochromes in the regulation of photoperiodism in *P. fascia* suggesting the existence of another GL photoreceptor for photoperiodism in *P. fascia*. Cryptochromes are BL receptors that function as day-length sensors in *Arabidopsis* (Mockler et al., 2003). Although cryptochrome is a receptor that primarily absorbs BL, reduced cryptochrome has an absorbance spectrum that extends into the GL range (Lin et al., 1995a, b), suggesting that cryptochromes may perceive both BL and GL in *P. fascia*. Although homologous cryptochrome genes have been identified in brown algae, their physiological functions remain unknown.

In this study, we demonstrated that NB treatment with RL enhanced erect thallus formation. Similarly, Lüning also reported that continuous irradiation with RL enhances erect thallus formation in *P. fascia* (1973). Some studies have reported BL-induced photomorphogenesis in brown algae (Dring and Lüning, 1975a, b, Lüning, 1981; Lüning and Dring, 1975; Wang et al., 2010; Yoshikawa et al., 2014); however, studies on RL-induced photomorphogenesis are rare (Duncan and Foreman, 1980; Lüning, 1973; Müller and Clauss, 1976). RL photoreceptor phytochrome-mediated photoresponse, which is also observed in terrestrial plants, is cancelled by FR irradiation shortly after RL irradiation (Thomas and Vince-Prue, 1997). In brown algae, only one response, potentially involving phytochromes, has been reported. FR induces stipe elongation in *Nereocystis luetkeana*, which is cancelled by RL irradiation (Duncan and Foreman, 1980). In this study, FR did not counteract the RL response in *P. fascia* (**Figure 5**). The response of *P. fascia* to RL may be a different type of photoreceptor-mediated response from that of *N*. *luetkeana.*

The phytochrome gene has been identified in *E. siliculosus* genome (Cock et al., 2010; Rockwell et al., 2014). As the spectrum of the recombinant protein of *E. siliculosus* phytochrome shows RL and GL photoconversion (Rockwell et al., 2014), we investigated whether the effects of GL are counteracted by RL irradiation immediately after GL irradiation and vice versa in this study. Notably, only the effects of GL were observed in experiments with GL irradiation, regardless of whether it was performed before or after RL irradiation. This result suggests that photoreceptors undergoing photoconversion with RL and GL are not involved in erect thallus formation in *P. fascia*. When GL irradiation preceded RL irradiation, RL did not promote erect thallus formation. This suggests that RL-induced erect thallus formation is not involved in day length perception but rather acts as a promoter under SD. Therefore, RL photoreceptors may function downstream of BL and GL receptors for day-length perception.

In conclusion, this study revealed that *P. fascia* uses both BL and GL for photoperiodic regulation, showing higher sensitivity to BL for erect thallus formation under SD conditions. RL further promotes erect thallus formation in NB treatment under SD conditions. Overall, this study provides insights into the photoperiodic strategies of brown algae from a physiological perspective and establishes a basis for future molecular biological studies on photoreceptor mechanisms.

## Data Availability

The original contributions presented in the study are included in the article/**Supplementary Material**. Further inquiries can be directed to the corresponding author.
