# Supplementary figures and images for: Night-break treatment with blue and green lights regulates erect thallus formation in the brown alga Petalonia fascia (KU-1293)
Source: Front Plant Sci. 2025 Jan 15;15:1500947. doi: 10.3389/fpls.2024.1500947 (PMC11774855; doi:10.3389/fpls.2024.1500947)

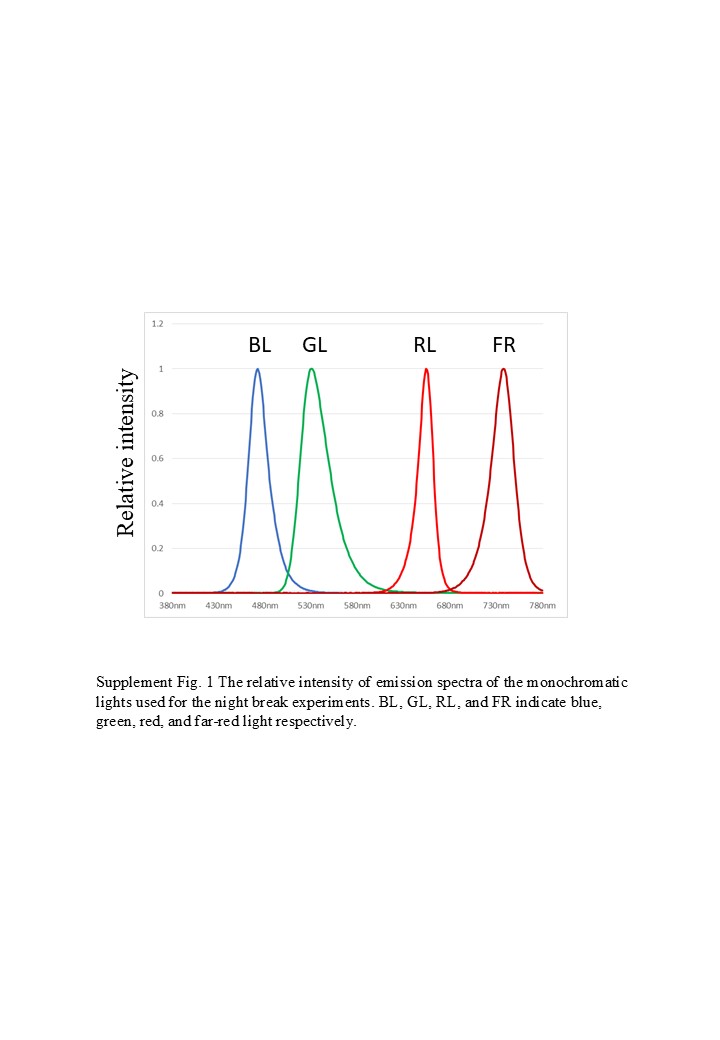

Supplement: Supplementary file 1 [file Image1.jpeg]

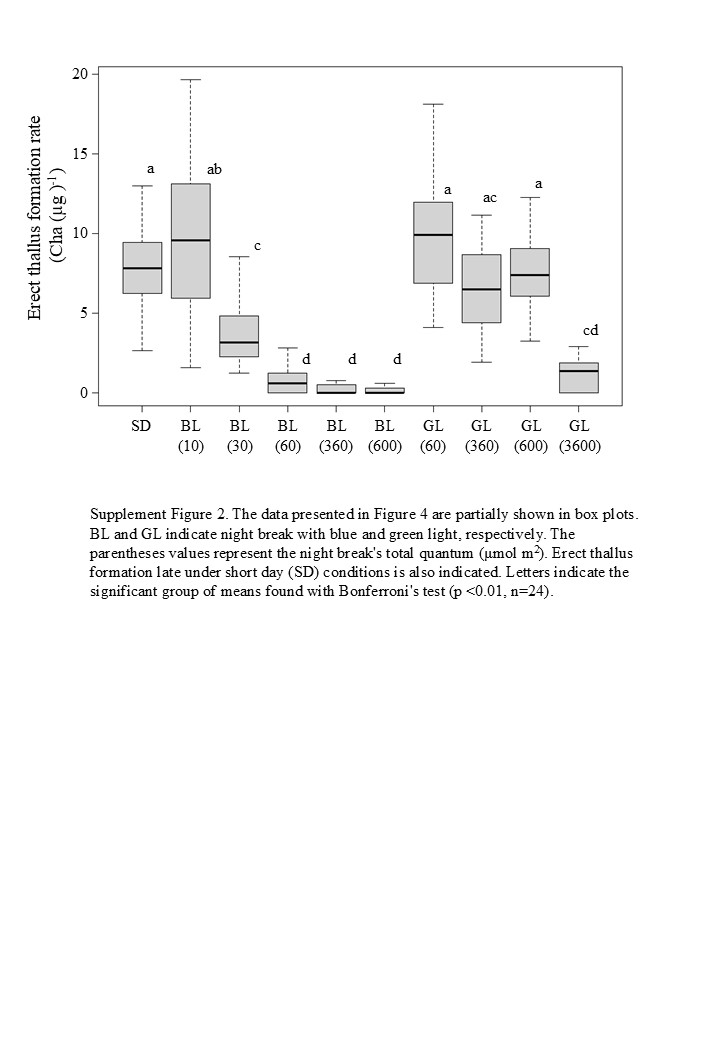

Supplement: Supplementary file 2 [file Image2.jpeg]

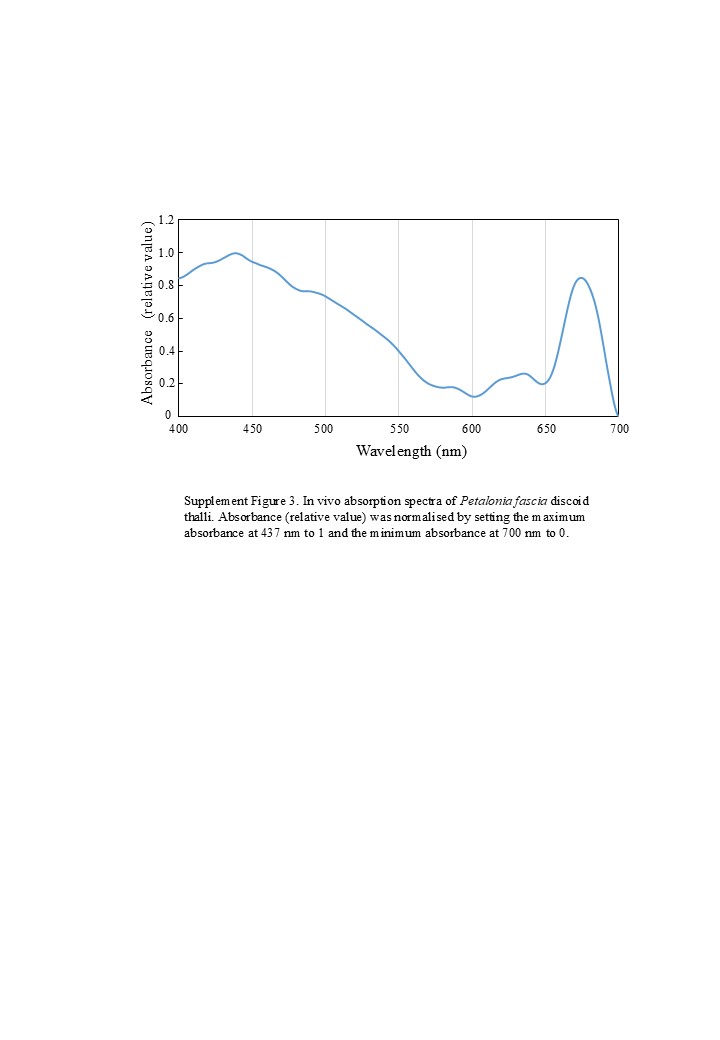

Supplement: Supplementary file 3 [file Image3.jpeg]

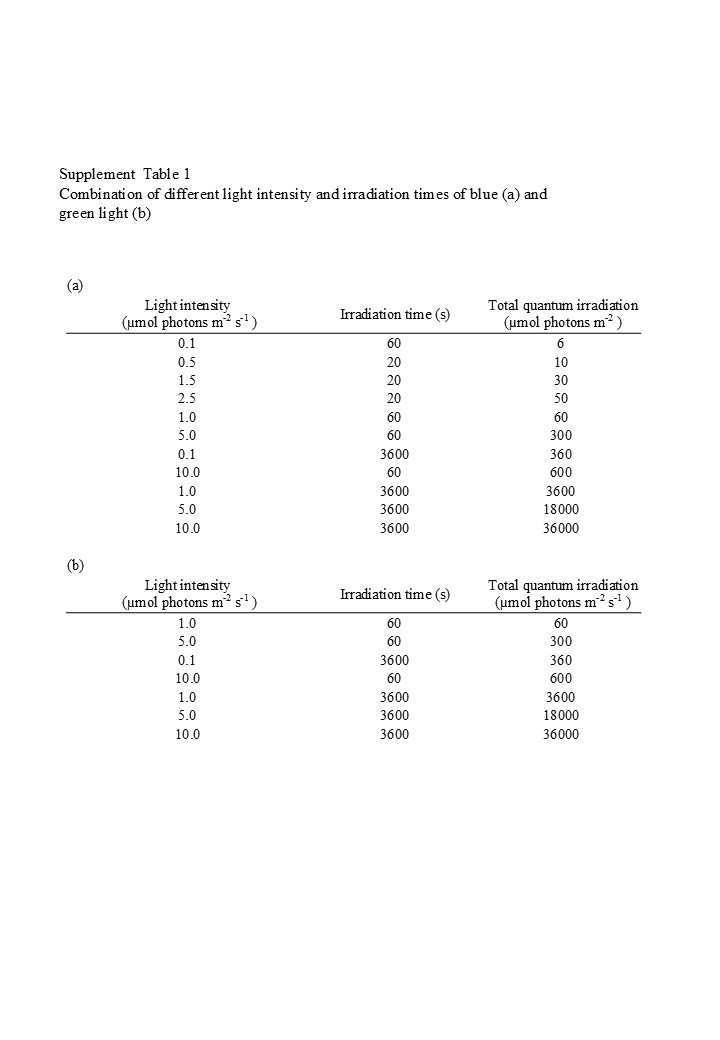

Supplement: Supplementary file 4 [file Image4.jpeg]
